# Influence of Alternative Prey on the Functional Response of a Predator in Two Contexts: With and without Intraguild Predation

**DOI:** 10.3390/insects15050315

**Published:** 2024-04-28

**Authors:** Lizette Cicero, Luis Enrique Chavarín-Gómez, Daniela Pérez-Ascencio, Ornella Barreto-Barriga, Roger Guevara, Nicolas Desneux, Ricardo Ramírez-Romero

**Affiliations:** 1 Laboratorio de Ecología Aplicada al Control Biológico (ECOBI), Instituto Nacional de Investigaciones Forestales, Agrícolas y Pecuarias (INIFAP), Mexico City 04010, Mexico; cicero.lizette@inifap.gob.mx; 2 Laboratorio de Control Biológico (LabCB-AIFEN), Departamento de Producción Agrícola, CUCBA, Universidad de Guadalajara, Guadalajara 44100, Mexico; chavarin.gomez@gmail.com (L.E.C.-G.); bugelle@gmail.com (D.P.-A.); barretoor@yahoo.com (O.B.-B.); 3 Red de Biología Evolutiva, Instituto de Ecología, A.C., Xalapa 91070, Mexico; roger.guevara@inecol.mx; 4 Université Côte d’Azur, INRAE, CNRS, 06903 Sophia-Antipolis, France

**Keywords:** intraguild predator, alternative prey, whitefly, *Geocoris punctipes*, *Eretmocerus eremicus*, *Myzus persicae*, parasitoid, pest control

## Abstract

**Simple Summary:**

The present study explores the impact of alternative prey on predator–prey dynamics in two contexts, without and with intraguild predation (IGP). It was found that without IGP, *G. punctipes* displayed a generalized functional response regardless of the alternative prey. However, under IGP conditions, the predator’s functional response shifted to Type II, but the alternative prey did affect this response and it turned to a generalized type. In both contexts, handling times increased and consumption of the focal prey decreased as the density of the alternative prey increased. The presence of parasitized whitefly nymphs notably affected predator–prey dynamics, probably due to changes in prey characteristics.

**Abstract:**

In biological control, joint releases of predators and parasitoids are standard. However, intraguild predation (IGP) can occur when a predator attacks a parasitoid, potentially affecting pest control dynamics. In addition to the focal prey (FP), *Trialeurodes vaporariorum*, the intraguild predator (IG-predator) *Geocoris punctipes* can consume the parasitoid *Eretmocerus eremicus* (IG-prey). In this IGP context with multiple prey, an alternative prey (AP), like the aphid *Myzus persicae*, may influence interactions. Theory predicts that, in simple interactions, a predator’s functional response (FR) to the FP changes with the presence of an AP. However, whether this holds in an IGP context is unknown. In this study, we empirically tested that prediction. Our results show that without IGP, *G. punctipes* exhibits a generalized FR with and without AP. Nevertheless, with IGP, the predator exhibited a Type II FR at low and high AP densities, increasing pressure on the FP and potentially favoring short-term biological control strategies. However, when 25 AP were offered, the predator’s response shifted, underscoring the importance of monitoring AP densities to prevent potential disruptions in FP control. In both contexts, the increase in AP produced a handling time increase and a decrease in consumption rate. These results indicate that the theoretical prediction of the effect of AP on the FR is met only under specific conditions, and the complexity of multitrophic interactions must be considered.

## 1. Introduction

The whitefly *Trialeurodes vaporariorum* Westwood (Hemiptera: Aleyrodidae) is a critical worldwide pest of several crops, including the tomato *Solanum lycopersicum* (Solanales: Solanaceae) [1]. This herbivore pest, referred to in this study as the focal prey (hereafter ‘FP’), can damage crops and reduce crop production by transmitting diseases, promoting fungal development, and extracting large amounts of sap [2]. Among the whitefly’s natural enemies, the big-eye bug *Geocoris punctipes* Say (Hemiptera: Lygaeidae) attacks whitefly nymphs and adults [3]. Also, the parasitoid *Eretmocerus eremicus* Rose and Zolnerowich (Hymenoptera: Aphelinidae) parasitizes whitefly nymphs [4]. However, interference may occur when natural enemies concur simultaneously [5,6,7,8]. For instance, intraguild predation (IGP) occurs when a predator preys upon parasitized nymphs of whiteflies, and competition occurs when a species limits the availability of prey for a second species [9,10]. In IGP, generally, the parasitoid becomes the prey (hereafter referred to as the ‘IG-Prey’) of the predator (hereafter referred to as the ‘IG-predator’) [11]. *Geocoris punctipes* have been reported to be an IG-predator of the parasitoid *E. eremicus* (IG-prey) [3,5], which can affect the demographic dynamics of the interacting species [9,10,11].

Under IGP contexts, an alternative prey (hereafter referred to as ‘AP’) could be present and modify the interactions among the IG-predator, the IG-prey, and the FP [6,12,13]. However, it is currently unclear whether the inclusion of an AP affects certain ecological aspects of the intraguild predator—such as its functional response—when a FP, an IG-prey, and an IG-predator are present simultaneously in intraguild predation contexts.

The functional response describes the relationship between the prey’s density and the number of prey that are attacked [14]. It plays a central role in the interactions among prey, parasitoids, and predators, as it influences changes in population dynamics [15,16]. In addition, it includes central parameters of predator–prey dynamics; therefore, it may be related to the success of one predator in biological control programs [17]. The more participants there are in a trophic network, the greater the number of possible complex and emerging responses, although predicting the underlying mechanisms and functional response is not straightforward [6,9].

Holling [18] describes three types of functional responses of predators preying upon their target prey. The type I response is expected when the FP is an herbivore, and the number of attacks by the predator is a linear function of prey availability. The type II functional response is characterized by an initial phase in which the number of attacked targets increases hyperbolically and then reaches an asymptote, reflecting the handling capacity of the predator. This response is the most common functional response in predator insects. Finally, the type III response found in insects, but mainly in vertebrates, describes a pattern where the rate of prey consumption rises slowly at low prey densities, accelerates rapidly as prey density increases, and then reaches a plateau at higher prey densities, illustrating a sigmoidal increase in attacked prey [19,20]. Subsequently, in 1977, Leslie A. Real [21] proposed an additional functional response representing a general type of sigmoidal response. This general type provides a more flexible and realistic representation of predator–prey interactions. This general type is called the general or generalized functional response [15,22]. The generalized functional response provides flexibility to fit better the various functional response patterns observed in predator–prey systems relative to types I, II, or III.

The theory predicts that in simple interactions, the presence of an AP will change the predator’s functional response to the FP because the presence of the AP distracts the predator’s attention from the FP [23,24]. Thus, proximal changes in the predator’s functional response due to an AP could affect the ecological interactions between the predator and its FP. For example, a change in the predator’s functional response from type II to type III due to the presence of an AP would prevent the predator from eliminating the FP of the ecosystem [25,26]. This hindrance could occur because the predation rate on the FP could decrease as the predator experiences satiety from consuming the AP [26,27,28,29]. From a biological control perspective, a decrease in FP consumption due to the presence of an AP can be considered, in the short term, as a negative impact [12]. However, in the context of simple interactions, few studies have empirically tested the effect of an AP on the predator’s functional response [30,31]. In addition, based on our literature search, that effect has not been tested in an IGP context.

We predict that *G. punctipes*, known for its type II functional response in basic predator–prey scenarios [32,33], could shift to a type III response when encountering an AP, as prey switching and preference could facilitate this shift [26,31].

On the other hand, it has been hypothesized within an IGP framework that the presence of an AP, serving as an additional resource for the IG-predator (as in our biological system), could render the FP, which hosts the IG-prey, less susceptible to predation and thus more likely to persist in the ecosystem [10]. In other words, with a more significant availability of the AP, the FP and the IG-prey consumption could be diminished [10]. The decrease in FP consumption could be reflected in a change in the predator’s functional response. Therefore, in the context of IGP, the prediction is also that *G. punctipes* will show a type II functional response when an AP is not present, and the functional response will change in the presence of an AP.

To test these predictions, our biological system included the whitefly *T. vaporariorum* as the FP and the aphid *Myzus persicae* (Sulzer) as the AP, together with the IG-predator, *G. punctipes* and the parasitoid *E. eremicus* (as the IG-prey). The whitefly *T. vaporariorum* is native to Central and South America, and its biology is well known [2]. The aphid *M. persicae* can colonize various crops, including tomatoes [34,35,36]. The IG-predator *G. punctipes* is native to America [37,38] and has a well-known biology [39]. The parasitoid *E. eremicus* is native to North America [40] and can effectively control the whitefly [41].

This study aimed to determine whether the theoretical prediction that a predator changes its functional response in the presence of an AP occurs in two contexts: with and without IGP.

## 2. Materials and Methods

All the plants and insects were grown and reared in separate chambers under environmental conditions of 24 ± 3 °C, 50 ± 10% relative humidity, and a photoperiod of 14:10 (light/darkness). The procedures for rearing and maintaining insects and plants were based on previous studies [3,5,6,41]. 

### 2.1. Plants

Tomato (*S. lycopersicum* c.v. Saladette) and pepper plants (*Capsicum annuum* L. c.v. Tampiqueño) (Solanales: Solanaceae) were used to rear and feed insect colonies and perform experiments. Tomato plants served as hosts for whiteflies, and peppers were used separately for rearing and feeding aphids. The seeds of both plant species were acquired in ‘La Casa del Hortelano S.A. de CV’ (Guadalajara, Jalisco, Mexico). For sowing, plastic trays (200 cavities and 7 cm deep) were used and filled with a substrate based on coconut dust acquired in a local market. One seed per cavity was sown. When plants reached two fully developed leaves, they were transferred into plastic pots (1.2 L of capacity). Those pots contained a mixture of soil (Nutrigarden^®^, Sulfatos y Derivados S. A. de C. V., Querétaro, Mexico) and perlite (Agrolita de México S. A. de CV, Estado de México, Mexico) in a 1:1 ratio. The plants were fertilized with ‘triple 18′ fertilizer (SQM Comercial de Mexico, SA de CV, Jalisco, Mexico). The plants were used when they reached between 4 and 5 development leaves. All plants were maintained free of herbivores before their use.

### 2.2. Whitefly Trialeurodes vaporariorum

The individuals of *T. vaporariorum* used for the experiments came from our colonies, which were started with individuals donated by Dr. Carla Sánchez-Hernández (University of Guadalajara, Zapopan, Jalisco, Mexico) whose taxonomic identification was verified by the specialist in Aleyrodidae, Dr. Vicente Carapia (Universidad Autónoma del Estado de Morelos, Cuernavaca, Morelos, Mexico). This colony of insects was reared in tomato plants inside acrylic cages (45 cm high × 38 cm long × 30 cm wide).

### 2.3. Aphid Myzus persicae

The aphids used in our experiments came from colonies started with individuals provided by Mary Carmen Torres Quintero (Universidad Autónoma del Estado de Morelos, Mexico). The aphid colonies were reared on pepper plants (*C. annuum* var. serrano) inside wooden cages (44 cm high × 44 long × 34 cm wide) covered with organdy. The Aphidoidea specialist Rebeca Peña Martínez (Instituto Politécnico Nacional, IPN, Mexico City, Mexico) verified the taxonomic identification. Aphids for experiments were randomly taken from the colonies when they reached the 3rd or 4th nymphal or adult stage.

### 2.4. Parasitoid Eretmocerus eremicus

Parasitoids were supplied by Koppert Mexico SA de CV (Querétaro, Mexico) as parasitized nymphs of *T. vaporariorum*. The parasitized nymphs were placed inside acrylic cages (45 cm high × 38 cm long × 30 cm wide). Once the adults emerged, they were fed a honey solution (2:1 mL; honey/water), which was offered on a paper towel (7 cm^2^) [42]. Wasps were also provided with tap water by moistening a paper towel (7 cm^2^) placed in a Petri dish (9 cm in diameter). Parasitoid females of *E. eremicus* were used when they were 2 to 6 days old [43].

### 2.5. Predator Geocoris punctipes

The *G. punctipes* individuals were provided as nymphs by Organismos Benéficos para la Agricultura SA de CV (Autlán, Jalisco, Mexico). Predator nymphs were kept in polystyrene cages (31 cm high × 40 cm long × 30 cm wide) and were fed an artificial diet [44], water (10 mL), pollen (5 g), and sorghum seeds (10 g) [45]. For experiments, female predators were used when they were 5 to 21 days old because they can live up to 70 days [39]. 

### 2.6. Parasitized Whitefly Nymphs

The procedure used to obtain parasitized whitefly nymphs (referred to as ‘parasitized-WN’) is described in detail by Velasco-Hernández et al. [3]. Overall, the leaves of tomato plants were enclosed in clip cages (6.4 cm in diameter × 3 cm high). In each clip-cage, 60 to 70 adult whiteflies were introduced and allowed to oviposit for 48 h. After this period, adult whiteflies were removed from the clip cage. Tomato plants were kept inside the rearing room, and about 15 days following whitefly oviposition, 2nd instar nymphs were obtained [42,46]. When 2nd instar nymphs were detected, seven pairs of *E. eremicus* were introduced per clip-cage and allowed to parasitize for 48 h. After this period, parasitoids were removed from the clip-cages. After 18–22 days, parasitized-WN were obtained. Parasitized nymphs, distinguished by their darker coloration compared to non-parasitized ones, were identified by visual inspection using a stereomicroscope (DV4, Carl Zeiss, Oberkochen, Germany).

### 2.7. Experiment 1: Effect of Alternative Prey on the Functional Response of G. punctipes without IGP

#### 2.7.1. Predator Starving

Before using the predators in the experiments, they were starved for 24 h to homogenize their appetites and stimulate their food search [32,47]. Predators were starved by placing them individually in a petri dish (9 cm in diameter) containing only water for 24 h.

#### 2.7.2. Experimental Arenas

The experimental arena was set up with a Petri dish (9 cm diameter) layered with filter paper atop a 5 mm agar base [3]. Within this setup was the designated number of whitefly nymphs alongside a pepper leaf placed to one side. Aphids (AP) were set on the pepper leaf as necessary. The whitefly nymphs were established in the arenas, as described in Velasco-Hernández et al. [3]. Briefly, leaf discs containing a single whitefly nymph were prepared by cutting tomato leaves hosting 2nd–3rd instar whitefly nymphs. Subsequently, the precise number of nymphs (leaf discs) was placed onto the filter paper for use in the bioassays [3]. The aphids were taken from the colonies and carefully added to the arenas using a soft brush on the pepper leaf. The setting of the arenas started one day before the beginning of the experiment by placing the whitefly nymphs. The next day, the aphids (AP) were introduced into the arenas when appropriate, just before the introduction of predators. To ensure the freshness of both the pepper leaf and leaf discs during the predation period, we did not provide aphids with a 1- or 2-day establishment period. The experiment started when a female predator was introduced into the arena.

In this experiment, the FP densities selected (i.e., 5, 10, 25, 50, and 80) to assess the functional response of *G. punctipes* were based on previous studies [32,33]. Additionally, from these FP densities, we chose densities representing low (5), medium (25), and high (80) levels of AP to evaluate its effects, consistent with the methodologies of prior studies [27,31].

In such a way, the functional response of the predator to the whitefly nymphs was assessed in the four alternative prey (AP) treatments: (1) without AP (from now on, referred to as ‘No AP’), (2) AP at a low density (from now on, referred to as ‘5 AP’), (3) AP at a medium density (from now on, referred to as ‘25 AP’), and (4) AP at a high density (after this, referred to as ‘80 AP’). Five experimental arenas were established for each treatment, with each arena containing an increasing number of whitefly nymphs, namely 5, 10, 25, 50, and 80. We followed a randomized block design for this experiment, considering the time (experimentation day) as the blocking factor. Each treatment was replicated six times, considering previous studies on the functional response [32,33]. New organisms were used for each replicate to avoid pseudo-replication.

After a female predator was released in the experimental arena, it was allowed to prey upon whitefly nymphs or aphids for 24 h, considering previous results [3]. After this period, the number of whitefly nymphs and aphids preyed upon in each experimental arena was recorded. The predator was removed from the testing arena, and nymphs were observed under a stereoscope (DV4, Carl Zeiss) to determine if they were preyed upon, following the method of Bao-Fundora et al. [5]. The functional response to each treatment (No AP, 5 AP, 25 AP, and 80 AP) was determined using the number of nymphs preyed upon at each arena (details in the statistical analysis section). In addition, the No AP treatment (i.e., only whitefly nymphs without AP) allowed the type of functional response of the predator in the absence of AP to be determined. Consequently, the other treatments assessed the effect of different AP densities on the predators’ functional response.

### 2.8. Experiment 2: Effect of AP on the Functional Response of G. punctipes When IGP Is Present

For this experiment, organisms, experimental conditions, and devices were like those described in Experiment 1. The main difference was that parasitized whitefly nymphs (prepared as described above) were used instead of the non-parasitized whitefly nymphs employed in Experiment 1. Experiment 2 allowed us to study the functional response in an IGP context, where the IG-predator *G. punctipes* consumes whitefly nymphs previously parasitized by *E. eremicus* (IG-prey). In this experiment, the functional response of the predator on parasitized WN was determined on four alternative prey (AP) treatments: (1) without AP (only parasitized WN and referred to as ‘No AP2’), (2) AP at a low density (parasitized WN and five aphids, referred to as ‘5 AP2’), (3) AP at a medium density (parasitized WN and 25 aphids, from now on referred to as ‘25 AP2’), and (4) AP at a high density (parasitized WN and 80 aphids, after this referred to as ‘80 AP2’). As in experiment 1, five experimental arenas containing an increasing number of parasitized WN for each treatment were established. The experimental design of this experiment was the same as that of Experiment 1, and each treatment was replicated six times, using new organisms per replicate. The functional response to each treatment (No AP2, 5 AP2, 25 AP2, and 80 AP2) was determined as indicated below.

### 2.9. Statistical Analysis

#### 2.9.1. Comparison of Consumed Whiteflies and Aphids

Generalized linear models (GLMs) were conducted to compare the number of consumed whitefly nymphs and aphids (AP) offered to *G. punctipes* for Experiments 1 and 2 (without and with IGP). The number of consumed whitefly nymphs (FP) and the number of consumed aphids (AP) were used as dependent variables. The independent variable was the whitefly density (5, 10, 25, 50, and 80 whitefly nymphs) for each AP treatment (No AP, 5_AP, 25_AP, and 80_AP) in the two experiments, without and with IGP (parasitized nymphs). Different families and link functions were tested to select the best model fit for each AP treatment. The results can be found in Table S1.

#### 2.9.2. Functional Response (FR) Analysis

Functional Response models are based on a general equation proposed by Holling [18,48]. These models describe how the prey consumption rates change due to the density of prey offered to a predator. Changes will depend upon the availability of prey and the time invested in the search, capture, and handling of prey. From this derives the type II FR equation modeled by Holling, known as the disc equation:

(1) $$N_{e}=\frac{aNT}{1+aNh}$$

where *N_e_* is the number of prey eaten, *a* is the rate of successful searches (attack rate), *T* is the total time available (experimental time), *h* is the handling time per prey, and *N* is the prey density.

However, Holling’s disc equation assumes that prey density does not decline over time. Therefore, in experiments with prey depletion, like this one, Rogers [49] proposed a modification that consists of integrating instantaneous consumption over time, obtaining the following equation:

(2) $$N_{e}=N_{0}\;(1-exp\;\left(a\;N_{0}^{q}\;\left(hN_{e}-T\right)\right))$$

*N*_0_ is the initial prey density, *q* is a scaling exponent, and the other parameters are as used in the previous equation.

On the other hand, the FR type III can be modeled using Equation (1), as long as the attack rate *a* is a hyperbolic function of the prey density (*a* = *bN*), where *b* is the attack coefficient. In this way, the functional response has a sigmoidal shape [50]. The equation for type III FR, integrating prey depletion, is as follows [51]:

(3) $$N_{e}=N_{0}\;\left\{1-exp\;\left[\left(d+b\;N_{0}\;\right)\left(T_{h}\;N_{e}-T\right)/(1+c\;N_{0}\right]\;\right\}$$

Although, in most cases, functional responses are described as type II or III, Real [21,52] proposes a general form. The generalized FR is a more flexible way of fitting FR models, incorporating the possibility of gradually shifting between types II and III [15,22]. In the adaptation of Real, the attack rate, *a*, depends on the density of the prey (*a* = *bNq*), leading to an FR model that can be written as follows:

(4) $$N_{e}=\frac{b\;N_{0}^{1+q}}{1+bh\;N_{0}^{1+q}}$$

where *q* is the scaling exponent that influences the shape of the FR, going from a decelerated hyperbolic curve to a sigmoidal one [22]. By allowing parameter *q* to take different values (*q* > 0), the model can capture a continuum of responses beyond the traditional type II (*q* = 0) and type III (*q* = 1) functional responses, capturing variations in feeding rates that simple functional response types cannot adequately describe.

We followed three steps to determine the type of FR for each treatment (in Experiment 1: No AP, 5 AP, 25 AP, and 80 AP and in Experiment 2: No AP2, 5 AP2, 25 AP2, and 80 AP2). The ‘frair’ package (version 0.5.100 [53]) of R statistical software [54] was used to fit all FR curves. The package is open development and available on GitHub (https://github.com/dpritchard/frair (accessed on 31 August 2023)). The first step was to determine the shape of the FR curve by fitting polynomial logistic regression models on proportional consumption data. According to Juliano [50], a type II FR is characterized by a negative linear term, contrasting with a positive linear term of type III. However, this approach does not consider the possibility of a generalized FR. For the second step, different models were fitted using the ‘frair_fit’ function, optimized with Maximum Likelihood Estimation (MLE), which was implemented using bbmle::mle2. We fitted and compared two models, one with a flexible *q* and one with a fixed *q* = 0, to distinguish between a type II FR or a sigmoidal. For each treatment, the best-fit model was selected based on the Akaike Information Criterion (AIC) (Table S2) recommended by Pritchard et al. [55]. Depending on the selected best model (with fixed *q* = 0 or flexible *q*), a Roger’s type II (if fixed *q* = 0 model was previously the best fit) or a Hassel’s type III (if flexible *q* model was previously the best fit) were fitted. If the flexible model performed better than the type III model, we concluded that a generalized response was the best fit. The starting values for the free parameters of each model were obtained from the raw data. We used *a* = 1 and *h* = 1/Fmax (where Fmax is the mean of consumed prey at maximum density), as recommended by Rosenbaum and Rall [22]. Thirdly, parameter confidence intervals (95%) were generated with the function ‘frair_boot’, which uses nonparametric bootstrapping and leverages boot::boot. Finally, maximum feeding rates (MFr = 1/hT) were calculated according to Pritchard et al. [55] for each treatment in both experiments.

## 3. Results

### 3.1. Prey Consumption among AP Treatments

**Figure 1:**
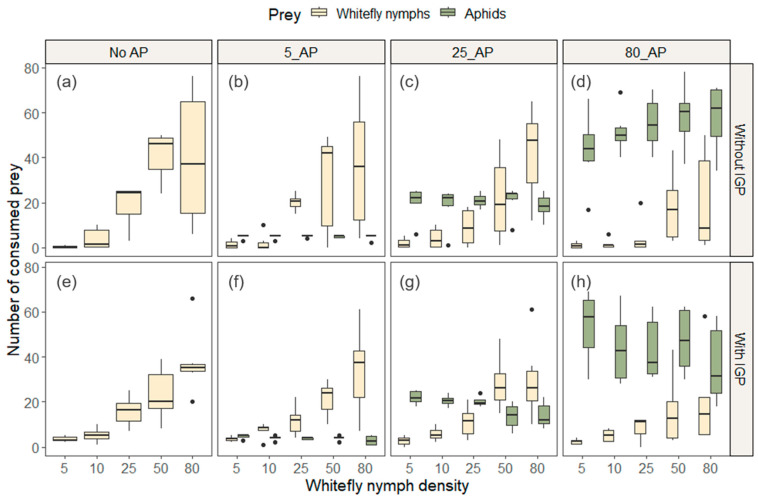
Number of consumed *T. vaporariorum* nymphs and *M. persicae* by *G. punctipes* across varying densities of *T. vaporariorum* nymphs within each alternative prey (*M. persicae*) treatment. Experiment 1, with non-parasitized whitefly nymphs (without IGP), (**a**–**d**), and Experiment 2, with parasitized whitefly nymphs (with IGP), (**e**–**h**).

The mean consumption of non-parasitized whitefly nymphs increased with the density of available nymphs across all treatments (Table 1, Figure 1a–d). However, whitefly consumption was slightly lower in the presence of 80 aphids (AP) (Table 1, Figure 1d) compared to treatments with fewer (Figure 1b,c) or no aphids (Figure 1a, Table 1 and Table S1). The maximum mean consumption of whitefly nymphs was 42 in the treatment with 25 AP (Figure 1c) at a nymph density of 80 (Table 1).

The predator’s mean consumption of aphids was relatively consistent across different nymph densities (Figure 1b–d and Table 1). However, when 80 aphids were available (Figure 1d), the predator consumed more than in treatments with fewer aphids (Figure 1b,c). The highest mean number of aphids consumed was 58, recorded in the treatment with 80 aphids and 50 whitefly nymphs (Table 1).

When parasitized whitefly nymphs were offered, an increase in whitefly density led to a corresponding rise in consumption (Figure 1e–h, Table 1 and Table S1). However, similar to the experiment without intraguild predation (IGP), when 80 AP were available (Figure 1h), whitefly consumption was slightly lower than in treatments with fewer AP (Figure 1f,g and Table S1). The highest mean number of consumed whitefly nymphs was approximately 38 in the treatment without AP (Figure 1e), at a density of 80 nymphs (Table 1). With IGP, aphid consumption remained similar, despite changes in nymph density when 5 and 80 aphids were offered (Figure 1f,h). However, aphid consumption decreased at high densities of parasitized whitefly nymphs when 25 aphids were available (Figure 1g). This observation suggests that, as the quantity of parasitized nymphs increases, the predator’s ability to consume aphids diminishes at this specific aphid density.

### 3.2. Functional Response Models

**Figure 2:**
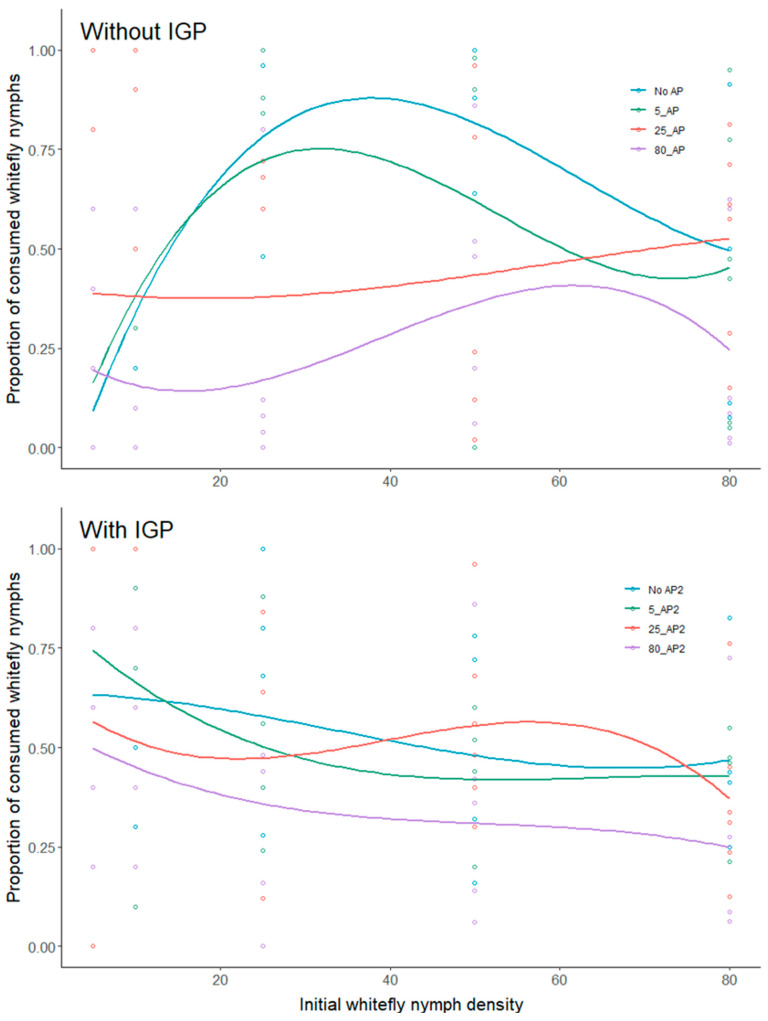
The proportion of *T. vaporariorum* nymphs consumed by *G. punctipes* at different densities of whitefly nymphs with four treatments of alternative prey (*M. persicae*). Experiment 1 involved non-parasitized whitefly nymphs (without IGP (**top**)), and Experiment 2 utilized parasitized whitefly nymphs (with IGP (**bottom**)).

**Figure 3:**
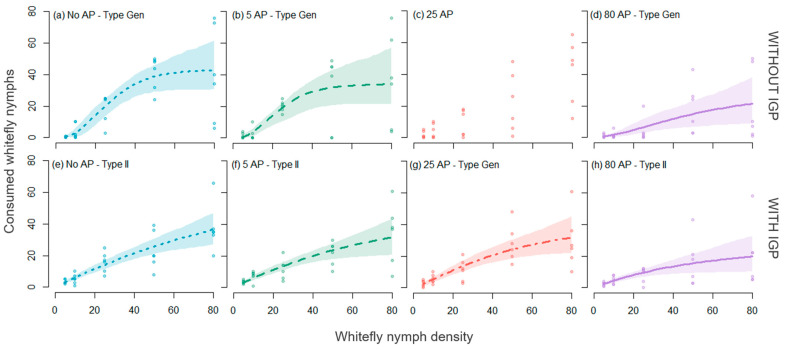
Functional response of *G. punctipes* preying on different densities of *T. vaporariorum* nymphs with four treatments of alternative prey (AP) *M. persicae*. Experiment 1 involved non-parasitized whitefly nymphs (without IGP), (**a**–**d**), and Experiment 2 utilized parasitized whitefly nymphs (with IGP), (**e**–**h**).

In Experiment 1 (without IGP), the polynomial logistic regression analyses of prey consumption proportions revealed a possible Type III FR across all four treatments, as indicated by the linear coefficients in Table 2, with the curve approaching an asymptote in a sigmoidal fashion (Figure 2 top). However, this initial analysis remains inconclusive, merely describing the overall shape of the response and discerning between hyperbolic and sigmoid curves (this approach is regarded as descriptive). Subsequent model fitting revealed that, for the No AP, 5_AP, and 80_AP treatments, the best-fitting FR model was the generalized model (Figure 3a–c), with the scaling parameter *q* not strictly limited to 0 (Type II) or 1 (Type III), but instead *q* > 0 (refer to Table 3 for the obtained *q* values). In contrast, none of the models in the 25_AP treatment showed a satisfactory fit with the significant parameter estimates. This outcome suggests that the predator’s consumption of whiteflies does not conform to tested functional response types, with precisely 25_AP. However, in this treatment, whitefly consumption increased with the increasing provision of whitefly nymphs (see Figure 1c).

Handling time (h) increased as more alternative prey were offered (Table 3). Accordingly, the maximum feeding rate (MFr) decreases from 44 prey/day in the treatment without AP to 32 in the 80 AP treatment (Table 3).

For the IGP experiment (with parasitized whitefly nymphs), the polynomial logistic regression analyses of the proportion of consumed prey indicated evidence of Type II FR in all four treatments (Table 2). This result suggests that the number of consumed prey hyperbolically approaches an asymptote as prey density increases (Figure 2 bottom). Upon model fitting, it was found that for the No AP, 5_AP, and 80_AP treatments, the best-fitting FR model was Type II, while for the 25_AP treatment, the best fit was with a generalized FR model (Table 3 and Figure 3e,f,h). Like the experiment without IGP, the handling time (h) increased as more alternative prey were offered, while MFr decreased from 96 prey/day to 35 (Table 3).

## 4. Discussion

The present study aimed to determine whether an alternative prey modified a predator’s functional response in contexts without and with IGP (i.e., whitefly nymphs parasitized by *E. eremicus*). We predicted that the predator’s functional response would change in the presence of an AP. However, this expectation was only partially met in the context of IGP, when 25 AP were presented, but not with fewer or more AP. Additionally, an increase in AP lowered the maximum feeding rates and increased the handling times of the predator (Table 3). In our study, *G. punctipes* preying upon whitefly nymphs without alternative prey and no IGP exhibited a generalized functional response.

### 4.1. Functional Response of G. punctipes without Alternative Prey and No IGP

Our results indicate that in a context without IGP and in the absence of AP (i.e., treatment No AP), *G. punctipes’* functional response to nymphs of *T. vaporariorum* is of the generalized or flexible type, which does not correspond with previous findings of a functional response for this predator. For instance, Cohen and Byrne [32] observed a type II functional response of *G. punctipes* when feeding adults of the whitefly *Bemisia tabaci* (Hemiptera: Aleyrodidae). Similarly, Parajulee et al. [33] found a type II functional response for *G. punctipes* to eggs of *Helicoverpa zea* (Lepidoptera: Noctuidae). However, these previous studies only focused on the three classic types of FR and did not assess whether the predation rates could align with the generalized functional response model [21,52]. Since the generalized functional response represents a feeding behavior between type II and type III curves, it is crucial to incorporate it into future studies [56]. Additionally, other factors, such as the age of the predator and the type of prey, can influence the functional response [33]. In our study, we used the nymphal stages of *T. vaporariorum* as prey, while Cohen and Byrne [32] used adults of *B. tabaci*. Subsequent studies that determine to what extent the species and the whitefly’s stage of development influence the predator’s functional response will help assess the influence of these factors on the predator’s feeding behavior.

### 4.2. Effect of Alternative Prey on the Functional Response of G. punctipes

#### 4.2.1. Without IGP

Theory predicts that, in the context of simple predator–prey interactions (i.e., without IGP), the functional response of a predator will change in the presence of AP [18,23,24]. For example, Real [52] found that a change in the ecological settings (i.e., alternate food and spatial distribution of prey) altered the general trend of an organism’s functional response. Studies on predator–prey scenarios (without IGP) have also reported a decrease in the consumption of the FP when AP is added. For example, Koss et al. [13] found that the predator *Geocoris* sp. decreased its consumption rate of the target prey, *M. persicae*, when the AP *Leptinotarsa decemlineata* (Coleoptera: Chrysomelidae) was added. Similarly, Bompard et al. [29] found that the mirid *Macrolophus pygmaeus* Rambur (Hemiptera: Miridae) decreased its consumption rate of the whitefly *(B. tabaci*) in the presence of the AP *Tuta absoluta* Meyrick (Lepidoptera: Gelechiidae). In line with these findings, our results showed that, in the context of no IGP, *G. punctipes,* the maximum feeding rate (Mfr) values decreased as more AP were added. However, the functional response of *G. punctipes* was of a generalized type, both in groups with and without AP. The functional response type did not change, although the FP consumption decreased when an AP was added.

#### 4.2.2. With IGP

When we analyzed the functional response in an IGP context (with parasitized whitefly nymphs), we found an AP effect on the predator’s functional response. The functional response was type II in all AP treatments, but when 25 aphids were provided, the predator exhibited a generalized functional response. This result indicates that with low densities of AP, the predator will show similar feeding dynamics as when there is no AP. However, upon reaching a certain number of AP (25 in our case), the predator will change its consumption dynamics and, therefore, the functional response. Some possible explanations could be related to the predator’s learning or prey preferences [21], changes in predator/prey proportions [15], or the defensive mechanisms of some prey, such as those related to immune responses [46,57].

Additionally, when the number of alternative prey becomes very high (80 in our study), the predator returns to its original consumption dynamics, probably because it refocuses on a single prey (see Figure 3g,h). Future studies are warranted to determine if these factors mediate these changes in consumption dynamics. Finally, it is worth noting that the persistence of the type II functional response against parasitized nymphs would imply that the intensity of IGP will likely be maintained even in the presence of alternative prey, confirming previous findings [6].

### 4.3. Functional Response of G. punctipes with and without IGP

#### 4.3.1. Absence of Alternative Prey (AP)

We observed distinct functional responses in simple predator–prey interactions (i.e., without IGP) compared to those in the IGP context (generalized and type II, respectively). This observation indicates that the parasitized state of the nymphs influences the predator’s functional response, transitioning from a quicker feeding dynamic (sigmoid curve) on non-parasitized nymphs to a slower one (hyperbolic curve) on parasitized nymphs. This outcome may be linked to *G. punctipes*’ documented preference for unparasitized nymphs over their parasitized counterparts [3]. Alternatively, the predator might adjust its functional response in response to its detecting the presence of the competing parasitoid within the whitefly nymph [58]. This hypothesis is grounded in the established understanding that factors such as competition [59], prey composition [60], and quality [61] can exert an influence on the functional response. Nevertheless, the likelihood of this scenario appears improbable as a prior study failed to uncover evidence indicating that the predator could differentiate between parasitized nymphs generating immune compounds and those that did not [46].

#### 4.3.2. Presence of Alternative Prey (AP)

In our study with non-parasitized whitefly nymphs (without IGP), predators exhibited a generalized (sigmoidal) functional response. In this response, the prey consumption rate increases slowly at low prey densities, accelerates rapidly as prey density increases, and reaches a plateau at high prey densities. This functional response occurred in our experiment, irrespective of the presence or absence of aphids (AP). Since this functional response has been linked with predators’ prey-switching behavior towards AP [26,62,63], our results suggest that with non-parasitized whitefly nymphs, the predator might reduce its consumption of nymphs when AP is present. This finding is consistent with previous research indicating that an AP can disrupt the biological control of certain FP species [6,28,64]. Type III and generalized functional responses are characterized by a sigmoid curve, where organisms such as vertebrates and insects can learn [19,20]. This learning capacity could relate to the exponential consumption phase before the inflection point and a decrease in consumption. Predators with a sigmoid functional response use prey according to their abundance, switching to the most abundant prey. Therefore, predator-switching behaviors can increase the population of prey with lower densities [17,65,66]. van Leeuwen et al. [16] explained that sigmoid functional responses like type III responses are more likely when the density of one prey species is constant or super-abundant while that of another prey changes. In our case, this applies only to the context of non-IGP.

In contrast, in our experiment with parasitized nymphs (with IGP), predators exhibited a Type II functional response characterized by increasing (hyperbolic) consumption of the FP at both low and high AP densities. However, with 25 AP, the predator altered this predation pattern to a sigmoidal response. Thus, our results suggest that the predator could be susceptible to switching prey to AP only at specific AP densities when parasitized nymphs are present.

In both contexts, when we analyzed the treatment with a medium AP density (i.e., 25_AP treatment), it appeared to destabilize FP consumption. No functional response model was fitted successfully in the first scenario (i.e., without IGP). This lack of fit may be due to the high variability of predators’ consumption of the focal prey produced by the medium number of alternative prey. Additionally, in the IGP scenario, we observed a change in the type of functional response from Type II to the sigmoid generalized type. This change could occur in response to the more stable amount of AP, as found by van Leeuwen et al. [16].

### 4.4. Relevance for Biocontrol Programs

Understanding insect predators’ functional responses is crucial for devising effective biological control strategies [12,17]. Different functional responses (e.g., Type I, Type II, Type III) govern the interactions between predator and prey populations, significantly influencing the outcomes of pest control efforts [20,57,61,67]. Characterizing insect predators’ functional responses is fundamental for developing sustainable biological control strategies [56,59].

The selection of the most suitable functional response type for biological control programs has been a subject of previous discussion. The appropriateness of a particular type may vary depending on the ecological context [52], whether this is a simple system like monocultures in greenhouses or a more complex environment such as natural ecosystems like forests. Type III functional responses are often favored in complex environments due to their potential for stabilization [68]. This preference is rooted in the adaptability and efficiency offered by a Type III response, characterized by a gradual increase in predation rate with increasing prey density, followed by a peak and eventual decline at higher prey densities [52]. This adaptive behavior is particularly advantageous in dynamic environments like open agricultural settings, where prey populations fluctuate significantly. A Type III response enables predators to regulate their feeding rate based on prey availability, ensuring adequate prey consumption to suppress pest populations without depleting prey resources [69]. By exhibiting this functional response, biological control agents can effectively manage pest populations, prevent outbreaks, and contribute to the sustainability of biocontrol programs. Conversely, Type II functional responses may lead to unstable predator–prey dynamics, posing risks of prey extinction at low densities and ineffective prey control at high densities. Type II functional responses are better suited for short-term, direct pest reduction programs, such as insect predator releases in seasonal crops [70].

Our research has practical implications, indicating that the species involved, the density of the AP, and predator preferences could significantly influence the efficacy of biological control predators against target pests. For example, in our study system, the absence of parasitoids (resulting in higher densities of non-parasitized nymphs) would reduce predator-mediated control of whiteflies in the presence of aphids (generalized functional response), potentially promoting a balanced ecosystem and favoring a conservation biological control approach. Conversely, when parasitoids were abundant and parasitized nymphs were present, predators consistently targeted the parasitized whiteflies (Type II functional response), exerting more significant pressure on the FP, which aligns with previous findings [6] and could favor short-term augmentative biological control strategies. However, our results indicate the importance of monitoring AP population densities, as predators may switch prey at certain thresholds, transitioning from a Type II to a generalized FR, potentially compromising control of the FP. These findings emphasize the importance of tailoring biological control strategies to particular ecological contexts to enhance their effectiveness and sustainability. For instance, investigating the influence of predator preference on functional response is essential. If the FP population falls below the economic injury level (EIL), utilizing a non-preferred AP in greenhouses could sustain the predator without jeopardizing its survival, and the predator could quickly revert to targeting the preferred pest prey. These additional studies hold the potential to offer valuable insights for refining pest management interventions.

### 4.5. Shortcomings and Prospects

We conclude that the theoretical prediction that AP will modify the predator’s functional response holds only in a complex context of predator–prey interactions (i.e., with IGP) and under particular circumstances (e.g., when given 25 aphids). Then, it is essential to highlight that the theoretical prediction will not always occur, since the predator will probably be able to respond based not only on the abundance of the AP but also on the characteristics (i.e., quality, mobility, and species) of the prey. While the AP did not alter the type of functional response in our experiment with non-parasitized whitefly nymphs (without IGP), it did influence both the magnitude of the response and the quantity of FP consumed. From a biological control point of view, our results indicate that the control exerted by the predator *G. punctipes* on non-parasitized whitefly nymphs could decrease as the AP (aphids) density increases. Furthermore, in parasitized whitefly nymphs, the AP could disrupt population dynamics by changing the functional response from hyperbolic to sigmoidal [68].

## Figures and Tables

**Table 1:** Mean and standard error of whitefly nymph and aphid consumption by G. punctipes per alternative prey (aphids) treatment, and for each experiment. Experiment 1 involved non-parasitized whitefly nymphs (without IGP), and Experiment 2 utilized parasitized whitefly nymphs (with IGP).

Experiment	Treatment	Prey Density	Consumed Whitefly Nymphs *	Consumed Aphids *	Total Consumed Prey
(1) Without IGP	No AP	5	0.33 ± 0.211 a		0.33
		10	3.83 ± 1.973 b		3.83
		25	19 ± 3.821 c		19
		50	41.17 ± 4.369 c		41.17
		80	39.67 ± 12.298 c		39.67
	5_AP	5	1.33 ± 0.715 a	4.67 ± 0.333 a	6
		10	2.17 ± 1.641 a	4.67 ± 0.333 a	6.84
		25	20.17 ± 1.4 b	4.83 ± 0.167 a	25
		50	29.67 ± 9.472 b	4.67 ± 0.211 a	34.34
		80	36.5 ± 11.927 b	4.5 ± 0.5 a	41
	25_AP	5	1.83 ± 0.872 a	20 ± 2.96 a	21.83
		10	4.17 ± 1.851 a	18.5 ± 3.62 a	22.67
		25	9 ± 3.464 ab	20.8 ± 1.22 a	29.80
		50	22 ± 7.698 bc	21 ± 2.66 a	43
		80	42 ± 8.327 c	18.5 ± 2.2 a	60.50
	80_AP	5	1 ± 0.516 a	43.3 ± 6.65 a	44.30
		10	1.5 ± 0.922 a	51.7 ± 3.96 a	53.20
		25	4.33 ± 3.169 ab	55.3 ± 4.76 a	59.63
		50	18.17 ± 6.426 b	58.3 ± 5.73 a	76.47
		80	19.67 ± 9.376 b	57.8 ± 6.19 a	77.47
(2) With IGP	No AP2	5	3.5 ± 0.5 a		3.50
		10	5.17 ± 2.676 a		5.17
		25	15.83 ± 2.676 b		15.83
		50	23.17 ± 4.888 bc		23.17
		80	37.67 ± 6.195 c		37.67
	5_AP2	5	3.5 ± 0.428 a	4.5 ± 0.342 a	8
		10	7.33 ± 1.333 ab	3.83 ± 0.401 a	11.16
		25	11.67 ± 2.654 bc	3.67 ± 0.211 a	15.34
		50	21.5 ± 3.096 cd	3.83 ± 0.401 a	25.33
		80	34 ± 7.894 d	2.83 ± 0.833 a	36.83
	25_AP2	5	2.67 ± 0.803 a	21.8 ± 1.19 a	24.47
		10	5.67 ± 1.202 ab	20.5 ± 0.992 a	26.17
		25	11.17 ± 2.821 b	20.2 ± 0.872 a	31.37
		50	28.17 ± 4.778 c	13.5 ± 2.31 b	41.67
		80	29.67 ± 7.191 c	14 ± 2.32 b	43.67
	80_AP2	5	2.33 ± 0.494 a	53.5 ± 6.33 a	55.83
		10	5 ± 1.125 ab	44 ± 6.6 a	49
		25	8.33 ± 2.076 abc	43.3 ± 5.77 a	51.63
		50	15.83 ± 6.263 bc	47.2 ± 5.9 a	63.03
		80	19.83 ± 8.308 c	36.5 ± 7.14 a	56.33

* Different letters within the columns of consumed prey (whiteflies and aphids) per AP treatment indicate significant differences.

**Table 2:** Results of polynomial logistic regression analysis of the proportion of whitefly nymphs eaten by *G. punctipes* for each alternative prey (aphids) treatment in both experiments. Experiment 1 involved non-parasitized whitefly nymphs (without IGP), and Experiment 2 utilized parasitized whitefly nymphs (with IGP).

Experiment	Treatment	Coefficient	Estimate	SE	*z* Value	*p* Value
(1) Without IGP	No AP	Intercept	0.038	0.1194	0.316	0.7518
		Linear	3.2579	0.6141	5.31	0.0000
		Quadratic	−5.7776	0.5659	−10.209	<0.0001
		Cubic	1.29764	0.54117	2.398	0.0165
	5_AP	Intercept	−0.9046	0.109	−8.3	<0.0001
		Linear	1.504	0.5768	2.608	0.0091
		Quadratic	−2.1778	0.4715	−4.619	<0.0001
		Cubic	1.3379	0.3849	3.476	0.0005
	25_AP	Intercept	−0.8954	0.07989	−11.21	<0.0001
		Linear	0.8066	0.34921	2.31	0.0209
	80_AP	Intercept	−1.6198	0.1276	−12.695	<0.0001
		Linear	1.6888	0.6298	2.682	0.0073
		Quadratic	−1.4268	0.4405	−3.239	0.0012
(2) With IGP	No AP	Intercept	−0.6082	0.0705	−8.632	<0.0001
		Linear	−0.5728	0.324	−1.769	0.0769
	5_AP	Intercept	−0.67628	0.07154	−9.453	<0.0001
		Linear	−0.73974	0.32983	−2.243	0.0249
	25_AP	Intercept	−0.6751	0.0724	−9.332	<0.0001
		Linear	−0.8125	0.3393	−2.394	0.017
	80_AP	Intercept	−1.0385	0.08074	−12.862	<0.0001
		Linear	−1.19816	0.38369	−3.123	0.00179

**Table 3:** The functional response of *G. punctipes* preying on whitefly nymphs, estimated parameters (a = attack rate, h = handling time, b = attack constant), and maximum feeding rates (MFr = prey/day) for each treatment of alternative prey (aphids) in both experiments. Experiment 1 involved non-parasitized whitefly nymphs (without IGP), and Experiment 2 utilized parasitized whitefly nymphs (with IGP).

Experiment	Treatment	FR Type	Parameter	Estimate	SE	z Value	*p* Value	95% CI	
								Lower	Upper
(1) Without IGP	No AP	Flex-gen	b	0.0028	0.0019	1.4369	0.1508	0	0.124
			h	0.0228	0.0008	27.7029	<0.0001	0.011	0.034
			q	2.1645	0.2289	9.4571	<0.0001	0.702	3.704
			MFr	44					
	5_AP	Flex-gen	b	0.0047	0.0037	1.2479	0.2121	0	0.446
			h	0.0289	0.0013	21.8089	<0.0001	0	0.05
			q	2.0329	0.2884	7.0486	<0.0001	0.14	3.536
			MFr	35					
	25_AP	No evidence of any functional response type							
	80_AP	Flex-gen	b	0.0293	0.0237	1.235	0.2168	0	0.373
			h	0.0314	0.0081	3.8826	0.0001	0	0.096
			q	0.7936	0.2701	2.9377	0.0033	−0.105	2.583
			MFr	32					
(2) With IGP	No AP2	Rogers-II	a	0.9797	0.1138	8.6089	<0.0001	0.634	1.632
			h	0.0104	0.0029	3.5373	0.0004	0	0.024
			MFr	96					
	5_AP2	Rogers-II	a	0.981	0.1264	7.7604	<0.0001	0.583	1.915
			h	0.015	0.0034	4.4635	<0.0001	0	0.039
			MFr	67					
	25_AP2	Flex-gen	b	0.445	0.2179	2.0423	0.0411	0.065	1.857
			h	0.0211	0.0046	4.5305	<0.0001	0	0.037
			q	0.2833	0.1864	1.5198	0.1286	−0.267	1.118
			MFr	47					
	80_AP2	Rogers-II	a	0.661	0.1033	6.4007	<0.0001	0.338	1.289
			h	0.0289	0.0060	4.8363	<0.0001	0	0.08
			MFr	35					

## Data Availability

The raw data supporting the conclusions of this article will be made available by the authors on request.

## Supplementary Materials

The following supporting information can be downloaded at: https://www.mdpi.com/article/10.3390/insects15050315/s1, Table S1: GLM results for whitefly nymph and aphid consumption among whitefly densities in both experiments, without and with IGP; Table S2: Akaike Information Criterion (AIC) values for the Functional Response Models of both experiments, without and with IGP.
