# Supplementary material for: Influence of Alternative Prey on the Functional Response of a Predator in Two Contexts: With and without Intraguild Predation
Source: Insects. 2024 Apr 28;15(5):315. doi: 10.3390/insects15050315 (PMC11122098; doi:10.3390/insects15050315)
Supplement: Supplementary file 1 [file insects-15-00315-s001.zip › Table S1.pdf]

Table S1. Generalized Linear Model analyses of the number consumed whiteflies and aphids, comparing among whitefly densities offered, inside each treatment of alternative prey (aphids) for Experiment 1 (without intraguild predation) and 2 (with intraguild predation using parasitized whitefly nymphs).

Experiment	Treatment	GLM family	Df	Deviance	Resid. Df	Resid. Dev	P
Consumed whiteflies among offered whitefly densities							
Without IGP	No AP	Negative Binomial	4	85.57	25	33.696	< 0.0001
	5_AP	Negative Binomial	4	30.477	25	35.37	< 0.0001
	25-AP	Negative Binomial	4	34.651	25	35.316	< 0.0001
	80_AP	Negative Binomial	4	26.165	25	32.094	< 0.0001
With IGP	No AP	Negative Binomial	4	108.12	25	28.359	< 0.0001
	5_AP	Negative Binomial	4	69.216	25	28.752	< 0.0001
	25-AP	Negative Binomial	4	79.94	25	31.722	< 0.0001
	80_AP	Negative Binomial	4	25.259	25	30.588	< 0.0001
Consumed aphids among offered whitefly densities							
Without IGP	5_AP	Gaussian	4	0.33333	25	16.333	0.9725
	25-AP	Poisson	4	1.8037	25	73.142	0.7718
	80_AP	Gaussian	4	912.13	25	4610.2	0.2929
With IGP	5_AP	Gaussian	4	8.5333	25	35.333	0.1963
	25-AP	Gaussian	4	371.33	25	416.67	0.0002
	80_AP	Negative Binomial	4	4.2927	25	30.497	0.3678
