# Supplementary material for: Influence of Alternative Prey on the Functional Response of a Predator in Two Contexts: With and without Intraguild Predation
Source: Insects. 2024 Apr 28;15(5):315. doi: 10.3390/insects15050315 (PMC11122098; doi:10.3390/insects15050315)
Supplement: Supplementary file 1 [file insects-15-00315-s001.zip › Table S2.pdf]

Table S2. Akaike Information Criterion (AIC) values for the Functional Response Models of both experiments, without and with IGP (parasitized whitefly nymphs). All models fitted were selected without prey replacement.

Experiment	Treatment	Fitted models	FRAIR package responses	AIC	Selected Model	Functional Response Type
1) Without IGP	*No AP*	Fixed *q*=0	flexpnr	735.3044	Flexible or Generalized	Type Gen
		Flexible *q*	flexpnr	594.2952		
		Type III *q*=1	hassIIInr	629.1702		
	5_AP	Fixed *q*=0	flexpnr	779.7787	Flexible or Generalized	Type Gen
		Flexible *q*	flexpnr	705.4561		
		Type III *q*=1	hassIIInr	776.7272		
	25_AP	No evidence of any response				
	80_AP	Fixed *q*=0	flexpnr	505.7692	Flexible or Generalized	Type Gen
		Flexible *q*	flexpnr	497.2479		
		Type III *q*=1	hassIIInr	501.9535		
2) With IGP	*No AP2*	Fixed *q*=0	flexpnr	306.1488	Roger's Type II	Type II
		Flexible *q*	flexpnr	307.7180		
		Type II *q*=0	rogersII	306.1488		
	5_AP2	Fixed *q*=0	flexpnr	312.1717	Roger's Type II	Type II
		Flexible *q*	flexpnr	338.6527		
		Type II *q*=0	rogersII	312.1717		
	25_AP2	Fixed *q*=0	flexpnr	348.5093	Flexible or Generalized	Type Gen
		Flexible *q*	flexpnr	348.2278		
		Type III *q*=1	hassIIInr	349.4491		
	80_AP2	Fixed *q*=0	flexpnr	398.8570	Roger's Type II	Type II
		Flexible *q*	flexpnr	400.3724		
		Type II *q*=0	rogersII	398.8570		
